# Exploitation of Biocomputational Approaches for siRNA‐Mediated Gene Silencing of HPV: A Novel Therapeutic Strategy for Cervical Cancer

**DOI:** 10.1002/cnr2.70434

**Published:** 2025-12-14

**Authors:** Md. Habib Ullah Masum, Rehana Parvin, Homaira Pervin Heema, Aklima Akter, Jannatul Ferdous

**Affiliations:** ^1^ Department of Genomics and Bioinformatics, Faculty of Biotechnology and Genetic Engineering Chattogram Veterinary and Animal Sciences University (CVASU) Bangladesh; ^2^ Department of Pathology and Parasitology, Faculty of Veterinary Medicine Chattogram Veterinary and Animal Sciences University (CVASU) Bangladesh; ^3^ Department of Obstetrics and Gynecology Chittagong Medical College Hospital Chattogram Bangladesh

**Keywords:** cervical cancer, gene silencing, *in silico*, siRNA

## Abstract

**Background:**

With recurrent high‐risk human papillomavirus (HPV) infections, especially HPV16 and HPV18, cervical cancer is the fourth most common disease among women. The functional alterations of the E6 and E7 proteins are essential to the oncogenic process of high‐risk HPVs and cervical cancer development.

**Aims:**

The objective of this research is to design highly targeted and efficient small interfering RNAs (siRNAs) that target both HPV16 and HPV18, employing cutting‐edge computational approaches to enhance therapeutic potential through a systematic bioinformatics‐driven approach.

**Methods and Results:**

This study utilized the i‐Score Designer to identify and evaluate four potential small interfering RNAs (siRNAs) (E6_69, E6_451, E7_66, and E7_193) based on various algorithm criteria, including Ui‐Tei, Amarzguioui, i‐Score, and Reynolds scores. Consequently, a docking analysis was conducted to elucidate the intermolecular interactions between the RNA‐induced silencing complex (RISC) proteins and the designed siRNAs. All siRNAs passed the recommended cutoff values, indicating a strong potential for gene silencing. Thermodynamic and structural analyses revealed that the designed siRNAs had favrable melting temperatures and free energies, suggesting adequate stability for effective gene silencing. Further, docking analysis demonstrated significant binding affinities and interaction profiles with the major RISC proteins (Dicer, Ago2, TRBP). The E6_69 and E7_193 significantly showed strong binding and intermolecular interactions, especially with Ago2, highlighting the potential for HPV gene silencing.

**Conclusion:**

The study revealed the potential of the designed siRNAs in silencing the E6 and E7 genes and their therapeutic applications against HPV. Future research should focus on validating these findings across various experiments, both in vivo and in vitro, and exploring alternative delivery approaches to enhance therapeutic efficacy.

## 1 Introduction

Cervical cancer is a lethal form of cancer that affects sexually active women and is more prevalent among women aged 15–44 years [1, 2]. It ranks as the fourth most common gynecologic cancer globally [3]. Chronic infections with high‐risk human papillomaviruses (HPVs) have been shown in epidemiological and clinical research to be a key cause of cervical cancer; HPV16 accounts for about 50% of cervical cancer cases associated with HPV [2]. HPV is a small (8 kb), nonenveloped, double‐stranded DNA virus classified under the Papillomaviridae family, responsible for benign cutaneous warts and juvenile respiratory papillomatosis, as well as serving as a precursor to cancer [4, 5]. The association between HPV and cervical cancer was first identified in the 1970s [6, 7]. However, HPV has been reported with other types of cancers, including cancers of the vulva, vagina, anus, and oropharynx [8, 9]. HPVs are classified as either high‐risk HPV or low‐risk HPV based on their ability to contribute to malignancies. HPV6 and HPV11 are typically considered low‐risk strains commonly associated with the development of benign anogenital warts [4]. The two primary types of HPV associated with high‐grade squamous intraepithelial lesions (HSIL) and cervical cancer are HPV16 and HPV18 [4, 10, 11].

According to a previous report, nearly 530 000 new cases of cervical cancer are identified annually, leading to about 270 000 fatalities worldwide [12]. In the year 2022, there were approximately 662 044 reported cases and 348 709 fatalities due to cervical cancer [13]. About 85% of these fatalities occur in underdeveloped or developing countries, where the mortality rate is 18 times higher than in developed countries [14]. Regions such as the Caribbean, Southern Asia, Sub‐Saharan Africa, and Central and South America have the highest incidence rates [14]. In the United States, the American Cancer Society reported an estimated 12 990 cases and 4120 deaths from cervical cancer in 2016 [15, 16]. However, each year, 11 500 new cases have been identified, with 4000 deaths [17]. Regarding cervical cancer, the HPVs are responsible for encoding the E6 and E7 oncogenes, the expression of which is also necessary for the virus's replication process [2, 18, 19]. The overexpression of E6 and E7 is responsible for developing malignant cells and is vital in sustaining the malignancy of cervical cancer [2, 20]. The overexpression of E6 and E7 is responsible for developing malignant cells and is vital in sustaining the malignancy of cervical cancer [1]. Therefore, these proteins disrupt the regulation of Plk1, leading to centrosome amplification and aneuploidy. The E6 protein is responsible for this disruption, resulting in the loss of p53, whereas the E7 protein leads to the loss of pRb family members, resulting in polyploidy [2, 21]. Additionally, both proteins can disrupt the gene regulation of cell cycle phases, such as G2 and M, which is crucial for mitosis, especially in centrosome balancing [22]. Currently, there is no authorized combined gene therapy for these proteins that could combat cervical cancer.

The development of small interfering RNAs (siRNAs) has been a significant breakthrough in the post‐genomic era. These remarkable molecules were initially observed in animals in 1998 and have since become a staple in functional genomics research [1, 23]. RNA interference (RNAi) is the process by which double‐stranded RNA molecules inhibit gene expression [1, 24, 25]. siRNAs can specifically target genes associated with several human disorders, including cervical cancer [1, 26]. RNAi therapies have shown great promise in regulating the expression of genes associated with human disease since their discovery in the 1990s [27]. In this type of gene therapy, Dicer, a protein of the RNA‐induced silencing complex (RISC), converts double‐stranded RNAs (dsRNAs) into siRNAs. These siRNAs direct other RISC proteins to cleave target mRNAs, such as by activating argonaute‐2 (Ago2) and transactivation response element RNA‐binding protein (TRBP) proteins [26]. Utilizing siRNA to target the E6 and E7 oncogenes of high‐risk HPV strains has shown considerable therapeutic potential in both in vitro and in vivo studies, especially in cervical cancer cell lines such as SiHa, CaSki, and HeLa [28, 29]. Early research focused on transient knockdown; however, subsequent approaches have highlighted sustained silencing, enhanced delivery techniques, and combinatorial therapy, including drugs such as cisplatin or wild‐type TP53 [28]. These advancements warrant the rationale for designing bioinformatics‐based siRNAs, specifically against HPV16 and HPV18, to enhance therapeutic efficacy and translational potential.

The precise regulation of gene expression allows for customized and tailored treatments for various diseases, thereby advancing the field of precision medicine [30, 31]. Traditionally, there has been much emphasis on research investigating siRNA design via sequence‐specific searches [32]. Therefore, computational‐based siRNA design is crucial for effectively addressing these drawbacks [30]. This study aimed to design a potential therapeutic siRNA using different computational algorithms and tools that might effectively combat cervical cancer by silencing the HPV oncogenes E6 and E7. This study stands out by utilizing a comprehensive, multi‐step computational approach for siRNA design, unlike previous studies that relied on singular tools or targeted only one HPV strain. This work combines various sophisticated computational approaches to facilitate the development of highly specific and effective siRNAs aimed at both HPV16 and HPV18. This dual‐strain approach, combined with a systematic bioinformatics‐driven process, substantially enhances the therapeutic significance of the findings and paves the way for novel targeted strategies against HPV‐related cancers.

## 2 Methods

**FIGURE 1:**
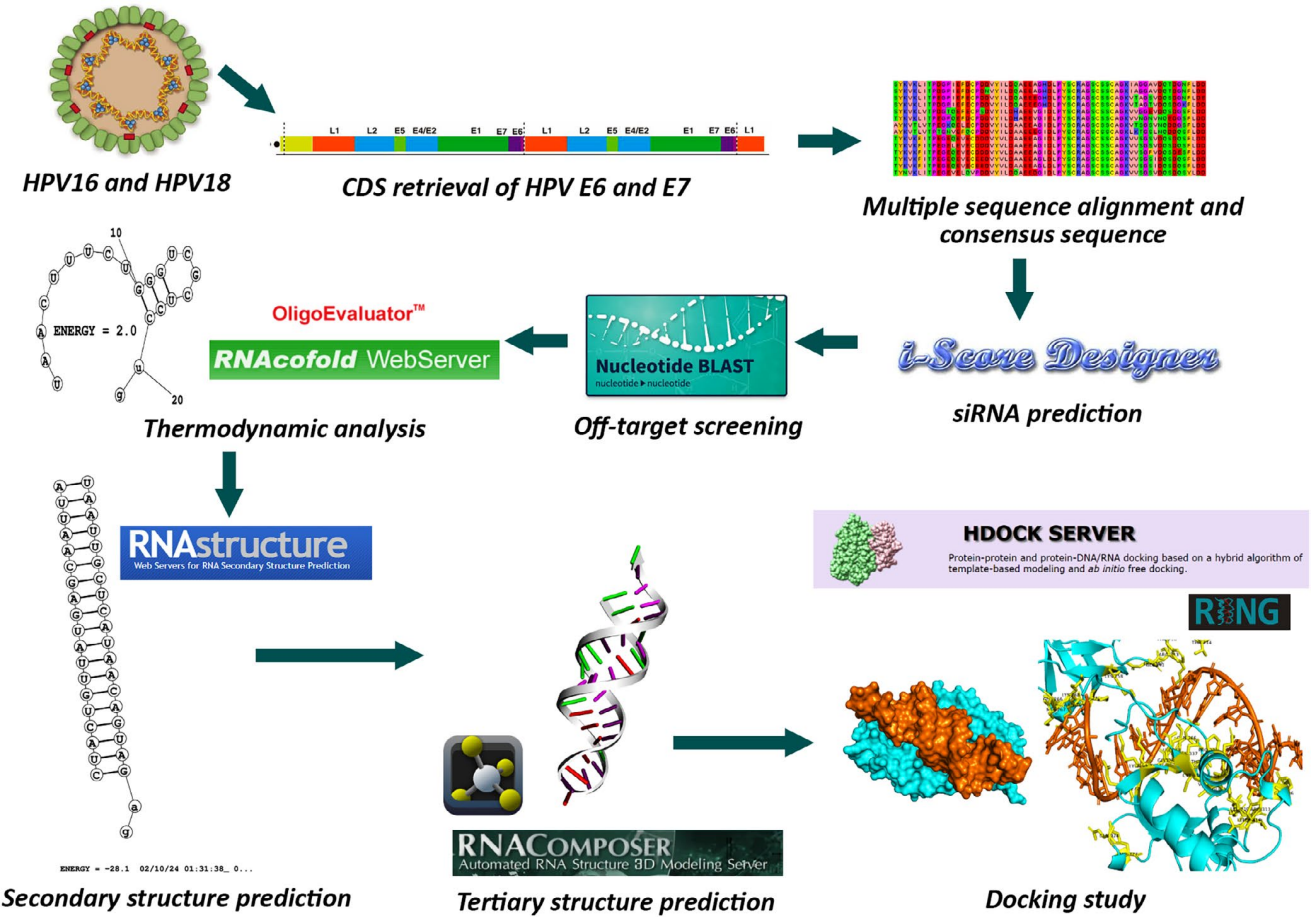
The overview of this study.

Figure 1 depicted the overview of this study.

### 2.1 Selection of Target Gene and Consensus Sequence Generation

The coding sequences (CDS) of the high‐risk HPV (HPV16 and HPV18); E6 and E7 genes were obtained from the National Center for Biotechnology Information (NCBI) nucleotide database. Afterwards, the MAFFT (version 7) online alignment program was used to generate the consensus and conserved sequences of the retrieved E6 and E7 CDS [33, 34]. Here, we applied the Clustal Omega algorithm to generate the conserved sequences of the selected genes using the multiple sequence alignment (MSA) approach. The output of this MSA, especially the consensus FASTA sequences (E6 and E7 each), was considered the representative sequences of the HPV E6 and E7 oncogenes. However, default parameter settings were used during the prediction process.

### 2.2 Prediction of siRNAs

The potential siRNAs against the HPV strains (HPV16 and HPV18) were predicted using the consensus sequences of the E6 and E7 through two factors evaluation: (1) sequence features and (2) target site accessibility [35, 36]. The i‐SCORE Designer web tool was used for sequence‐based siRNA design [37]. This tool evaluates several mRNA nucleotide preferences for siRNA prediction to calculate alternative algorithm scores, such as Ui‐Tei, Amarzguioui, Hsieh, Takasaki, s‐Biopredsi, i‐Score, Reynolds, Ka toh, and DSIR [35]. This study utilized rule‐based approaches, including the Ui‐Tei, Amarzguioui, and Reynolds scoring systems. For siRNA prediction using machine learning, the i‐Score algorithm was implemented. This algorithm utilizes a linear regression model to predict siRNA sequences [38]. However, GC content > 40% [39], Ui‐Tei rules (Ia or Ib) [40], Amarzguioui score > =3 [41], i‐Score > =66 [37], and Reynolds score > = 6 were considered threshold values for further siRNA screening [39].

### 2.3 Screening and Filtration of Off‐Target Sites

Identifying off‐target sites is essential in the siRNA design, especially for therapeutic uses, to ensure specificity and minimize unintended gene silencing. Off‐target effects arise when siRNAs exhibit partial complementarity to unintended mRNA transcripts, particularly in the seed region (positions 2–8 from the 5′ end of the guide strand), leading to suppression of non‐target genes via miRNA‐like mechanisms [42, 43, 44]. Non‐specific interactions may interfere with biological pathways, modify gene expression patterns, and induce toxic consequences, compromising the safety and efficacy of siRNA‐based therapies [45]. Filtering out siRNAs with high sequence similarity to off‐target transcripts or with strong thermodynamic pairing in the seed region reduces the risk of off‐target gene silencing [46]. A filtration approach was implemented through the NCBI nucleotide BLAST (https://blast.ncbi.nlm.nih.gov/Blast.cgi) program to minimize the risk of off‐target effects. Therefore, this was utilized to screen the human RefSeq mRNA database for perfect (19/19) or near‐perfect (18/19, 17/19) matches with both the sense (passenger) and antisense (guide) strands of the candidate siRNAs. This process ensured the specificity of the siRNAs to their intended targets by evaluating both the sense and antisense strands of the candidate siRNAs [47, 48]. Any siRNAs demonstrating complete (100% similarity) or near‐complete (≥ 78% similarity) complementarity with off‐target mRNA were excluded, so there should be no off‐target effects [49].

### 2.4 Thermodynamic Analysis

The melting temperature (Tm) of the sense and antisense strand for each potential siRNA duplex was determined using the OligoEvaluator analysis tool (http://www.oligoevaluator.com). The free energy of heterodimer binding (ΔG) between the sense and antisense strands of siRNA, the RNAcofold web server, was utilized with default parameters [50]. This application utilizes thermodynamic and kinetic parameters to compute interacting RNA strands' base‐pairing patterns and hybridization energy. The software also uses an enhanced version of McCaskill's partition function approach to calculate the base‐pairing probability [36, 51]. Each prospective siRNA duplex was subjected to minimal free energy (MFE) calculations to determine its structure within the ensemble and the ΔG value for heterodimer binding. The DINAMelt application was used to generate heat capacity and concentration plots for the predicted siRNAs [52]. The heat capacity plot illustrates the relationship between the collective heat capacity of the RNA duplex (Cp) and temperature. The Tm (Cp) is indicated by the peak of the heat capacity curve. Another detailed plot showcases the individual contributions of each species to the ensemble heat capacity. In contrast, the concentration plot illustrates the relative concentration of five species as the temperature changes. The Tm (conc) represents the temperature at which a double‐stranded molecule's concentration is half its maximum value [52].

### 2.5 Secondary (2D) Structure Prediction of siRNAs

Utilizing the MaxExpect algorithm, the RNA structure web server was applied to predict the secondary structure of siRNA and calculate the corresponding free energy [53]. Candidates with higher energy levels are more likely to be successful since the molecules involved are less likely to fold. A stronger sense‐antisense strand interaction also suggests a higher level of siRNA effectiveness. The DuplexFold tool was used to estimate the thermodynamic interaction between the sense and antisense siRNA strands [54].

### 2.6 Tertiary (3D) Structure Prediction of siRNAs

The 3D structures of the selected siRNAs were predicted using the RNAComposer server, which is publicly accessible [55]. This server obtains its information from the RNA FRABASE database, a search engine that is interoperable with the RNA tertiary structures database. Additionally, an approach based on motif templates is used to successfully predict complicated structures, such as multi‐branched loops and pseudo‐knotted loops [55]. The RNAalifold web server was utilized to predict the secondary structures of the designed siRNAs, providing results in dot‐bracket notation (Vienna format) [51]. These predictions were subsequently used as input for the RNA Composer web tool to generate detailed three‐dimensional models of the siRNA structures. The dot symbol used in dot‐bracket notation denotes the location of nucleotides that are not paired. Finally, the 3D configuration of the siRNAs was obtained by downloading it in the PDB file format.

### 2.7 Docking Analysis Between the Designed siRNAs and the Components of RISC‐Loading Complex

In this study, we performed a comprehensive series of molecular docking studies to evaluate the interactions between designed siRNAs and key proteins involved in the RISC‐loading complex. The proteins of interest in this study were Dicer, Ago2, and TRBP, all of which play crucial roles in the RNAi pathway. The crystallographic structures of these three proteins were accessible in the Protein Data Bank (PDB) with the PDB id 4NGF, 6RA4, and 5N8L for Dicer, Ago2, and TRBP, respectively [56]. Prior to docking analysis, molecules from these protein structures were removed and minimized by the Discovery Studio software [57]. Afterwards, the HDOCK server was applied for docking analysis [58]. The server facilitates the docking of protein–protein and protein‐DNA/RNA interactions by accepting amino acid sequences as input. It employs a sophisticated hybrid docking method that integrates experimental data on protein–protein binding sites and incorporates small‐angle X‐ray scattering (SAXS) data throughout the docking and post‐docking processes. This approach enhances the accuracy and reliability of the docking results, providing valuable insights into the structural and functional aspects of protein interactions [59, 60]. Finally, the RNA‐protein interactions were analyzed and visualized by the RING [61] and PyMol [62] tools, respectively.

## 3 Results

### 3.1 Prediction of siRNAs

Utilizing the conserved sequences of the E6 and E7 oncogenes, the i‐SCORE Designer web tool generated multiple siRNAs targeting the high‐risk HPV types (HPV16 and HPV18). Among them, four potential siRNAs, including E6_69, E6_451, E7_66, and E7_193, were chosen based on the recommended threshold for each of the five algorithms (GC content > 40%, Ui‐Tei Ia or Ib, Amarzguioui > = 3, i‐Score > =66, and Reynolds > = 6) (Table 1). Furthermore, these siRNAs were consistently ranked as the top candidates across all five predictive algorithms, above other predicted siRNAs. They demonstrated higher GC content and superior scores in key evaluation metrics, including Ui‐Tei Ia and Ib, Amarzguioui, i‐Score, and Reynolds criteria.

**TABLE 1:** List of predicted siRNAs with their five distinct algorithms.

SiRNA	Targeted gene	Sense	Antisense	% GC	Ui‐ Tei (Ia/Ib)	Amarzguioui (> = 3)	i‐ score (> = 66)	Reynolds (> = 6)
E6_69	E6	GGAGCGACCCAGAAAGUUA	UAACUUUCUGGGUCGCUCCug	52.6	Ia	4	80.4	7
E6_451	E6	GGUCGAUGUAUGUCUUGUU	AACAAGACAUACAUCGACCgg	42.1	Ia	4	69.6	6
E7_66	E7	CUACUGUUAUGAGCAAUUA	UAAUUGCUCAUAACAGUAGag	31.6	Ia	3	73.2	9
E7_193	E7	CUUCGGUUGUGCGUACAAA	UUUGUACGCACAACCGAAGcg	47.4	Ia	3	66.1	7

Here, the E6_69, having a GC content of 52.6% and a high Reynolds (7), i‐Score (80.4), and Amarzguioui (4) score, demonstrates great potential for gene silencing under the Ui‐Tei classification of Ia. The E6_451, with 42.1% GC content, was classified as Ia (Ui‐Tei) and provided Reynolds, i‐Score, and Amarzguioui scores of 6, 69.6, and 4, respectively (Table 1). However, the GC content (31.6%) of the E7_66 was below the threshold value and demonstrated gene silencing efficacy, as indicated by the Reynolds, i‐Score, and Amarzguioui scores of 9, 73.2, and 3, respectively. However, it was classified as Ia in terms of the Ui‐Tei parameter. Additionally, under the Ui‐Tei classification of Ia, the E7_193 showed a GC content of 47.4%, along with scores of 7, 66.1, and 3, respectively, using the Reynolds, i‐Score, and Amarzguioui algorithms (Table 1).

### 3.2 Thermodynamic Analysis

The stability of nucleotide base pairing is crucial in influencing the silencing mechanism of siRNA. The melting temperature and free energy change (ΔG) between siRNA seed and mRNA target are reliable indicators of the thermodynamic stability of these heteroduplexes. Thermodynamic data for four siRNAs (E6_69, E6_451, E7_66, E7_193) were assessed using OligoEvaluator, RNAcofold, and DINAMelt servers (Table 2). According to the OligoEvaluator, the sense and antisense strands of the E6_69 displayed two separate but satisfactory melting temperatures of 60.3°C and 62.9°C, respectively. Regarding the E6_451, the predicted melting temperatures for the sense and antisense strands are 53.6°C and 59.3°C, respectively. Meanwhile, the sense strands of the E7_66 had a Tm of 48.0°C, while the antisense strands had a melting temperature of 52.2°C. The E7_193 exhibited a Tm of 56.1°C and 52.4°C for the sense and antisense strands, respectively. As a result, it was discovered that all four siRNAs had internal melting temperatures below 65°C (Table 2).

**TABLE 2:** Thermodynamic properties of designed siRNA molecules.

siRNA		The OligoEvaluator	RNAcofold		DINAMelt					MaxExpect
		Tm°C	MFE (kcal/mol)	Heterodimer ΔG (kcal/mol)	Tm°C (Conc)	Tm°C (Cp)	ΔG (kcal/mol)	ΔH (kcal/mol)	ΔS (cal/mol/K)	Free folding energy (kcal/mol)
E6_69	GGAGCGACCCAGAAAGUUA	60.3	−35.80	−33.28	90.2	91.4	50.2	200	554.9	1.6
	UAACUUUCUGGGUCGCUCCug	62.9								2.0
E6_451	GGUCGAUGUAUGUCUUGUU	53.6	−32.20	−32.32	81.9	83.4	45.5	196.2	553.7	1.5
	AACAAGACAUACAUCGACCgg	59.3								1.9
E7_66	CUACUGUUAUGAGCAAUUA	48.0	−27.60	−26.23	80.3	82	40.3	181.6	519.8	1.5
	UAAUUGCUCAUAACAGUAGag	52.2								1.9
E7_193	CUUCGGUUGUGCGUACAAA	56.1	−31.90	−29.85	89.5	91.1	46.5	191.1	534.5	1.4
	UUUGUACGCACAACCGAAGcg	52.4								1.7

According to RNAcofold, the MFE scores of the E6_69, E6_451, E7_66, and E7_193 were predicted to be −35.80, −32.20, −27.6, and −31.90 kcal/mol, respectively. The heterodimer ΔG values of these siRNAs were found to be −33.28, −32.32, −26.23, and −29.85 kcal/mol, respectively (Table 2). The heat capacities of the duplexed siRNAs, as determined by DINAMelt results, were measured to be 90.2°C, 81.9°C, 80.3°C, and 89.5°C for the E6_69, E6_451, E7_66, and E7_193, respectively. The Tm of the E6_69, E6_451, E7_66, and E7_193 was determined to be 91.4°C, 83.4°C, 82°C, and 91.1°C, respectively. In addition, the ΔG values for the E6_69, E6_451, E7_66, and E7_193 were determined to be 50.2, 45.5, 40.3, and 46.5 kcal/mol, respectively. The values for enthalpy (ΔH) of the E6_69, E6_451, E7_66, and E7_193 were determined to be 200, 196.2, 181.6, and 191.1 kcal/mol, respectively. In addition, the entropy (ΔS) values for the E6_69, E6_451, E7_66, and E7_193 were determined to be 554.9, 553.7, 519.8, and 534.5 cal/mol/K, respectively (Table 2).

### 3.3 2D Structural Modeling of siRNAs

**FIGURE 2:**
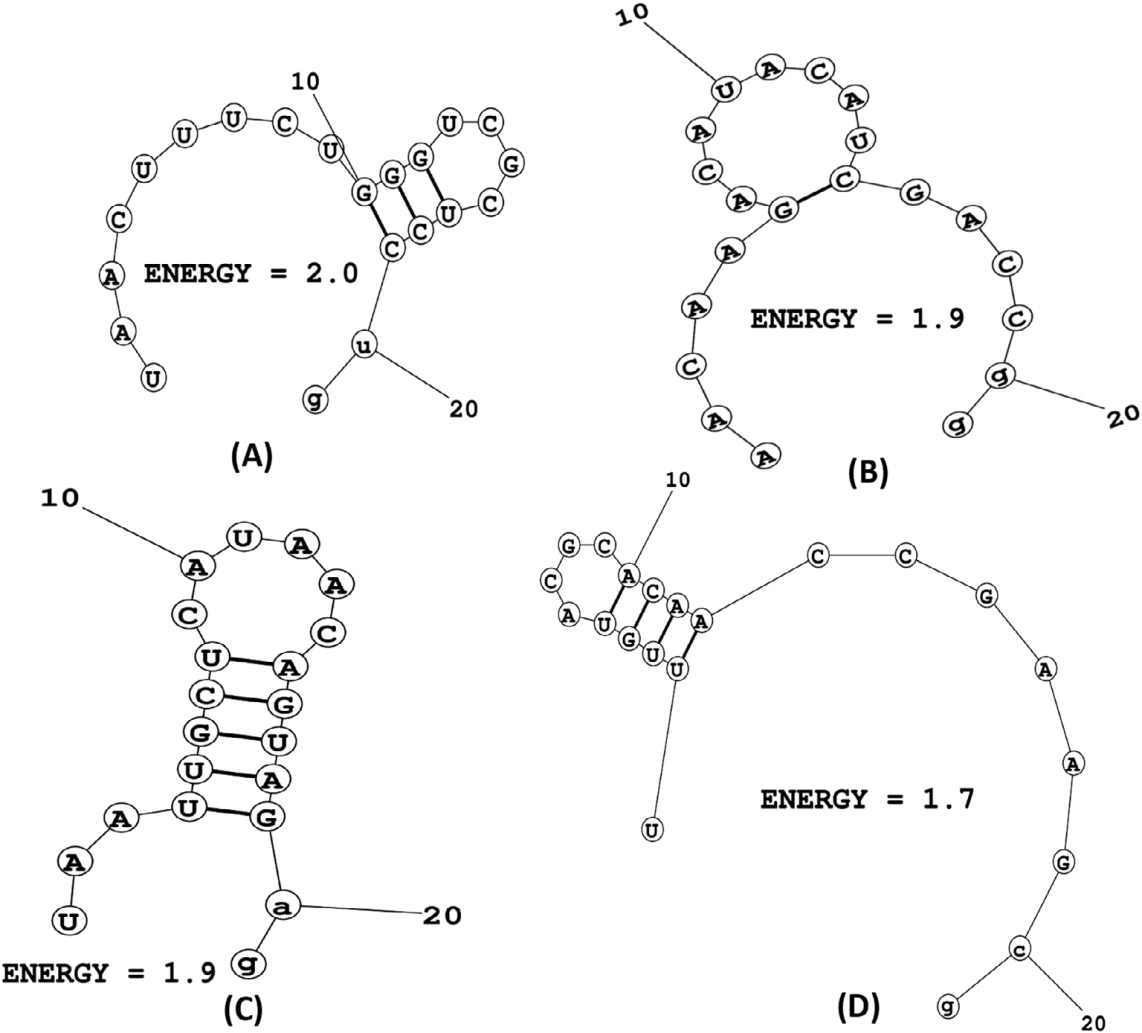
Thermodynamic secondary structures of the designed siRNAs, (A) E6_69, (B) E6_451, (C) E7_66, and (D) E7_193, with probable folding and lowest free energy for consensus sequence.

**FIGURE 3:**
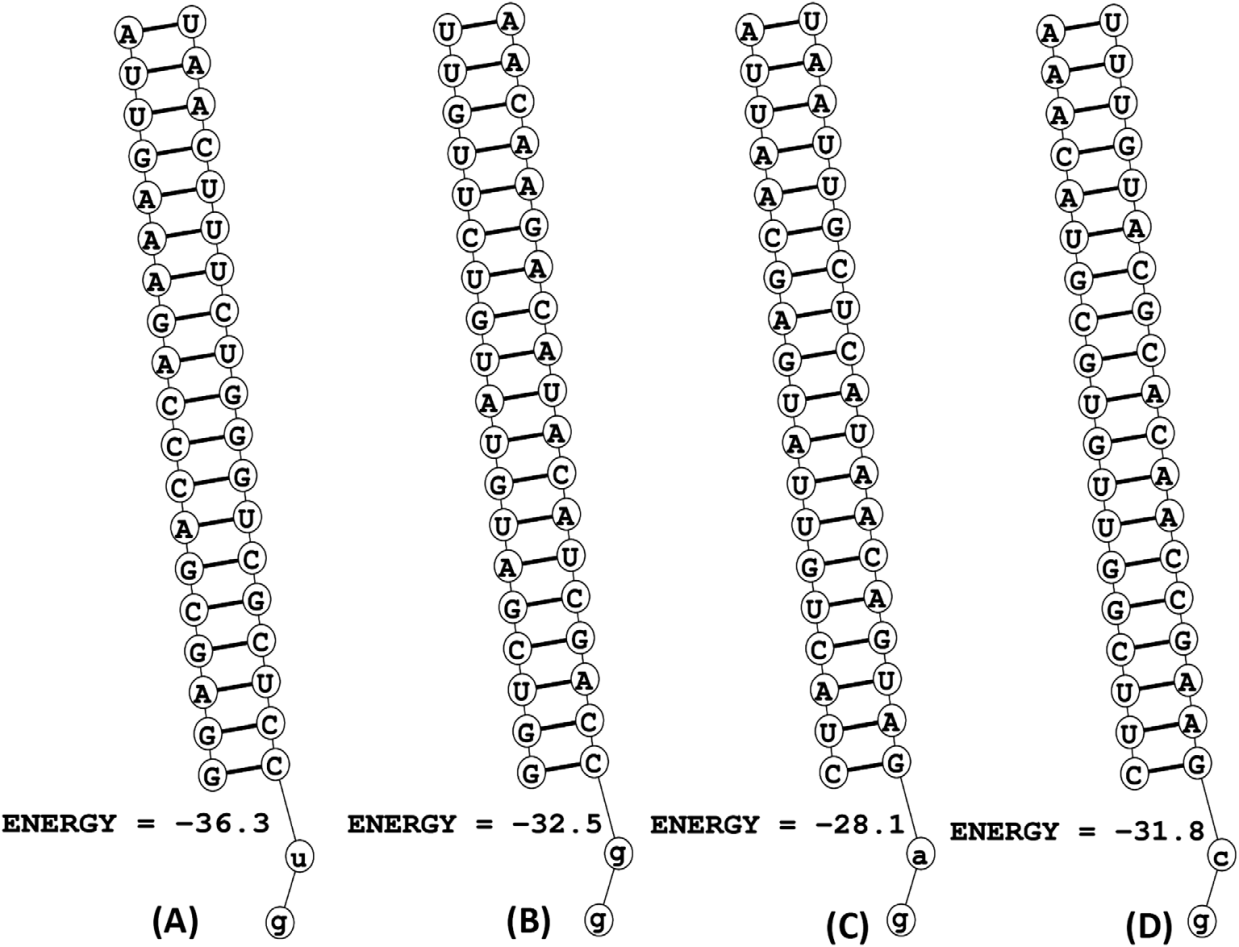
The Duplexfold predicted folding energies of the siRNAs with respective 2D representations, (A) E6_69, (B) E6_451, (C) E7_66, and (D) E7_193.

According to the MaxExpect tool, the calculated free energy of folding for the sense and antisense strands of the four siRNAs ranged from 1.4 to 2.0. Notably, the antisense strands exhibited higher folding energies compared to their sense counterparts. Among these, the antisense strand of E6_69 demonstrated the highest folding energy at 2.0, surpassing the antisense strands of E6_451 (1.9), E7_66 (1.9), and E7_193 (1.7) (Figure 2, Table 2). Furthermore, the Duplexfold server provided the folding energies for both sense and antisense strands. According to the server, E6_69 exhibited the highest accuracy with a folding energy of −36.3. In comparison, the folding energies for E6_451, E7_66, and E7_193 were −32.5, −28.1, and −31.8, respectively (Figure 3).

### 3.4 3D Structural Modeling of siRNAs

**FIGURE 4:**
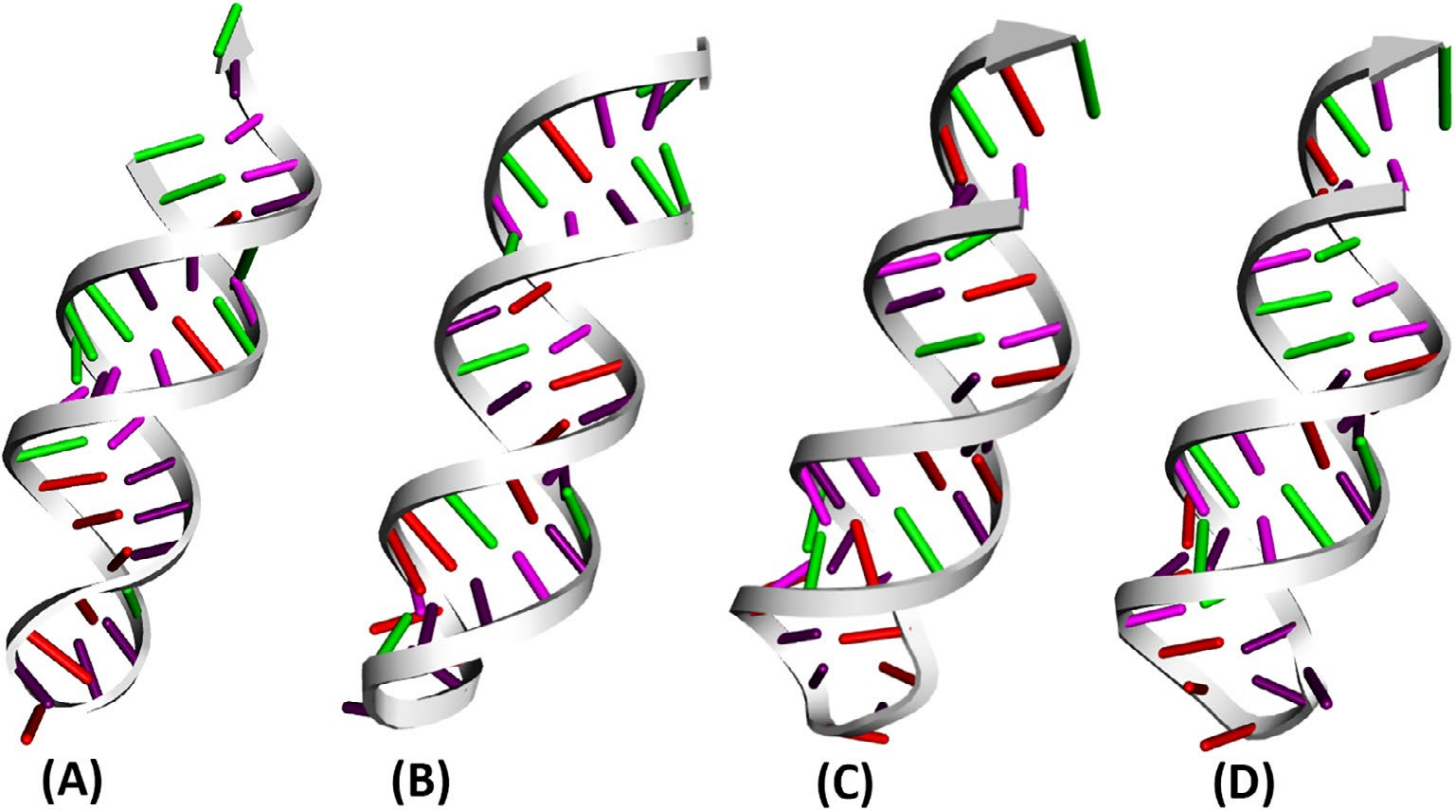
The predicted 3D structures of siRNAs, (A) E6_69, (B) E6_451, (C) E7_66, and (D) E7_193.

The predicted three‐dimensional structure of the chosen siRNAs was evaluated using the RNAComposer server (Figure 4). The execution of this intricate procedure unfolded in several phases, beginning with the fragmentation of secondary structures. This was succeeded by the preparation of 3D structure elements and rigid body transformations. Subsequently, the arrangement of these elements was optimized to ensure accuracy and precision.

### 3.5 Docking Analysis Between the Designed siRNAs and the Components of RISC‐Loading Complex

**FIGURE 5:**
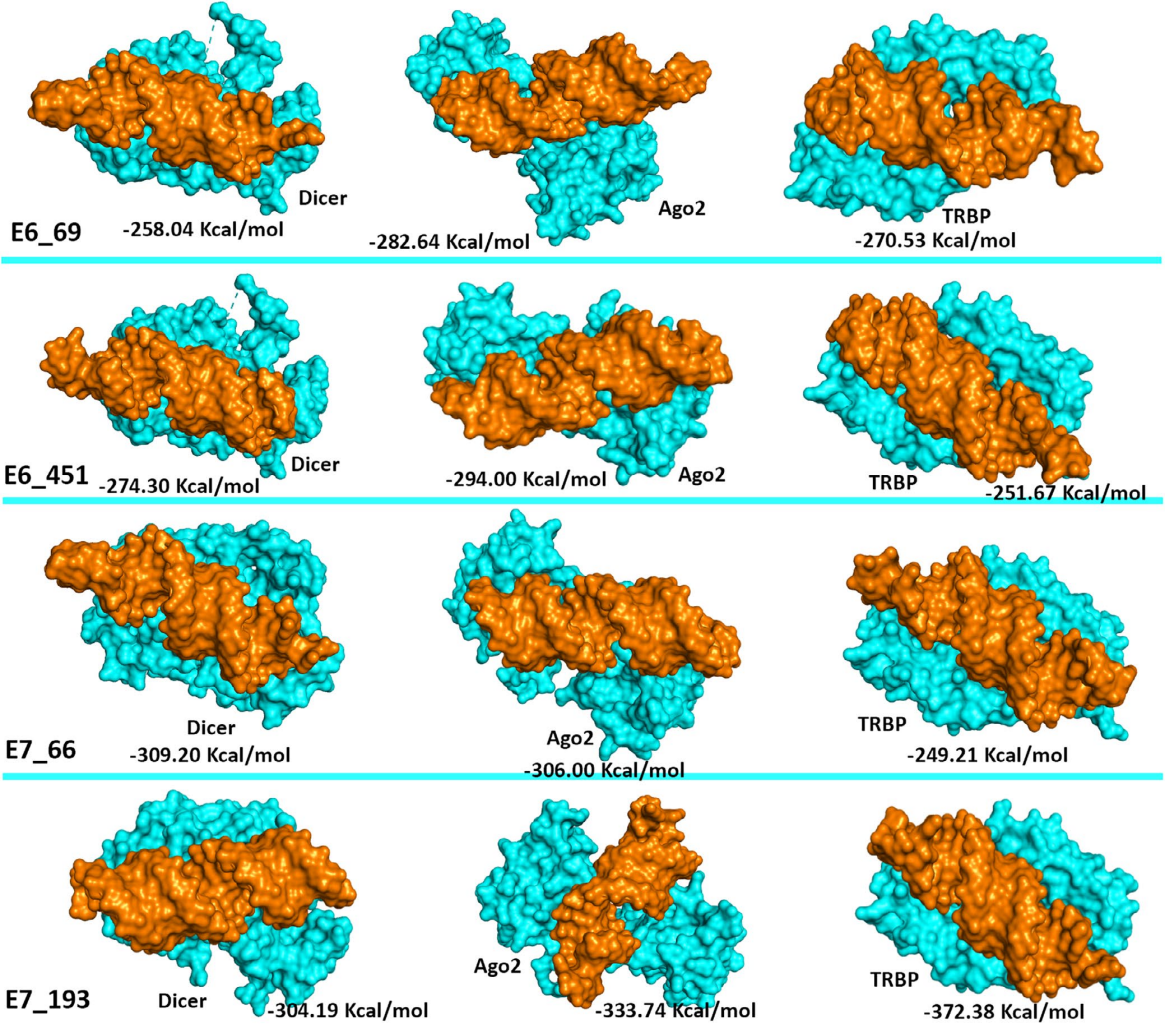
Docking analysis of the siRNAs and RISC complex components. The proteins are represented in cyan color, while the orange color represents the siRNAs.

**FIGURE 6:**
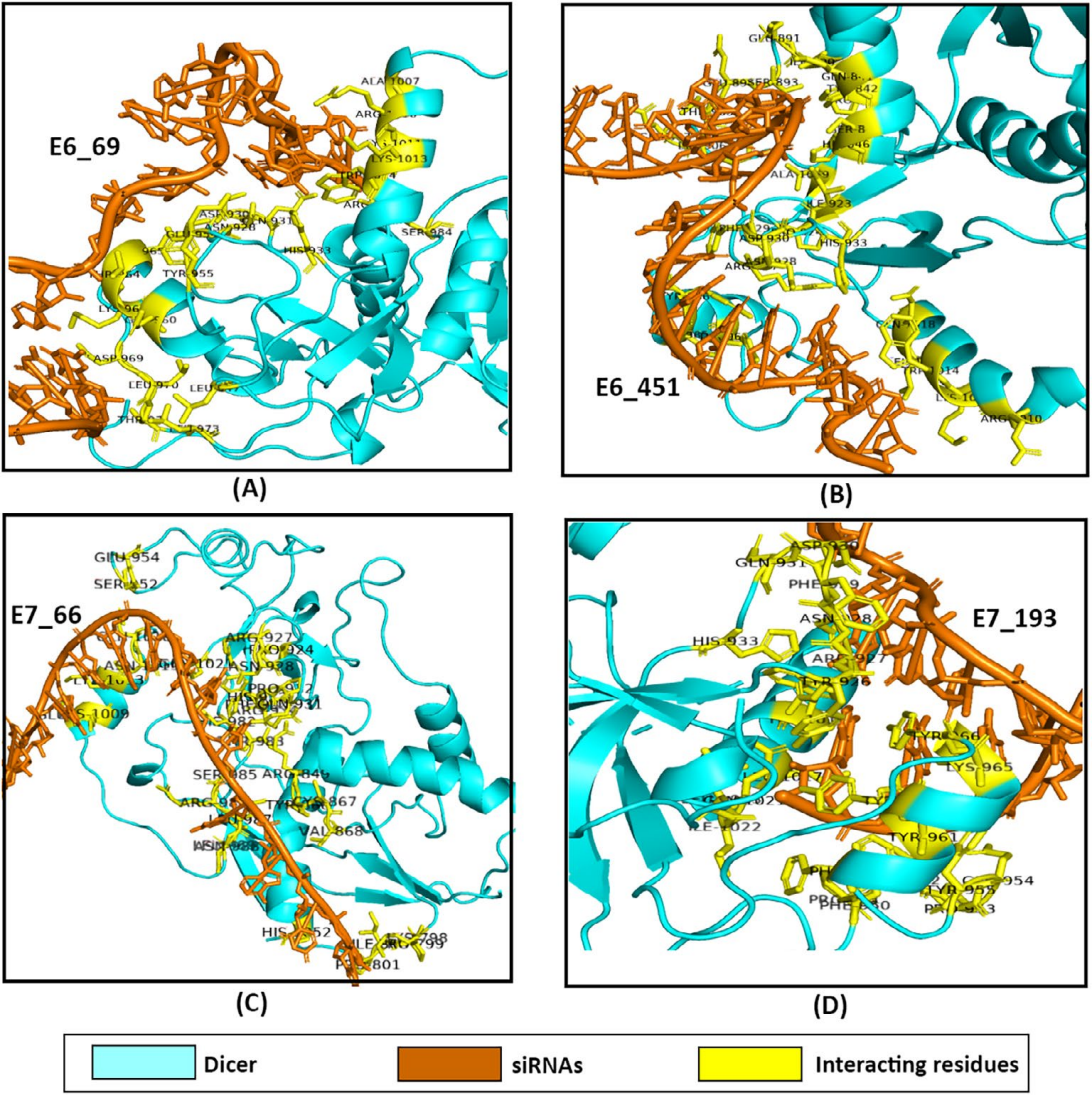
The protein‐siRNA interactions between (A) Dicer‐E6_69, (B) Dicer‐E6_451, (C) Dicer‐E7_66, and (D) Dicer‐E7_193.

**FIGURE 7:**
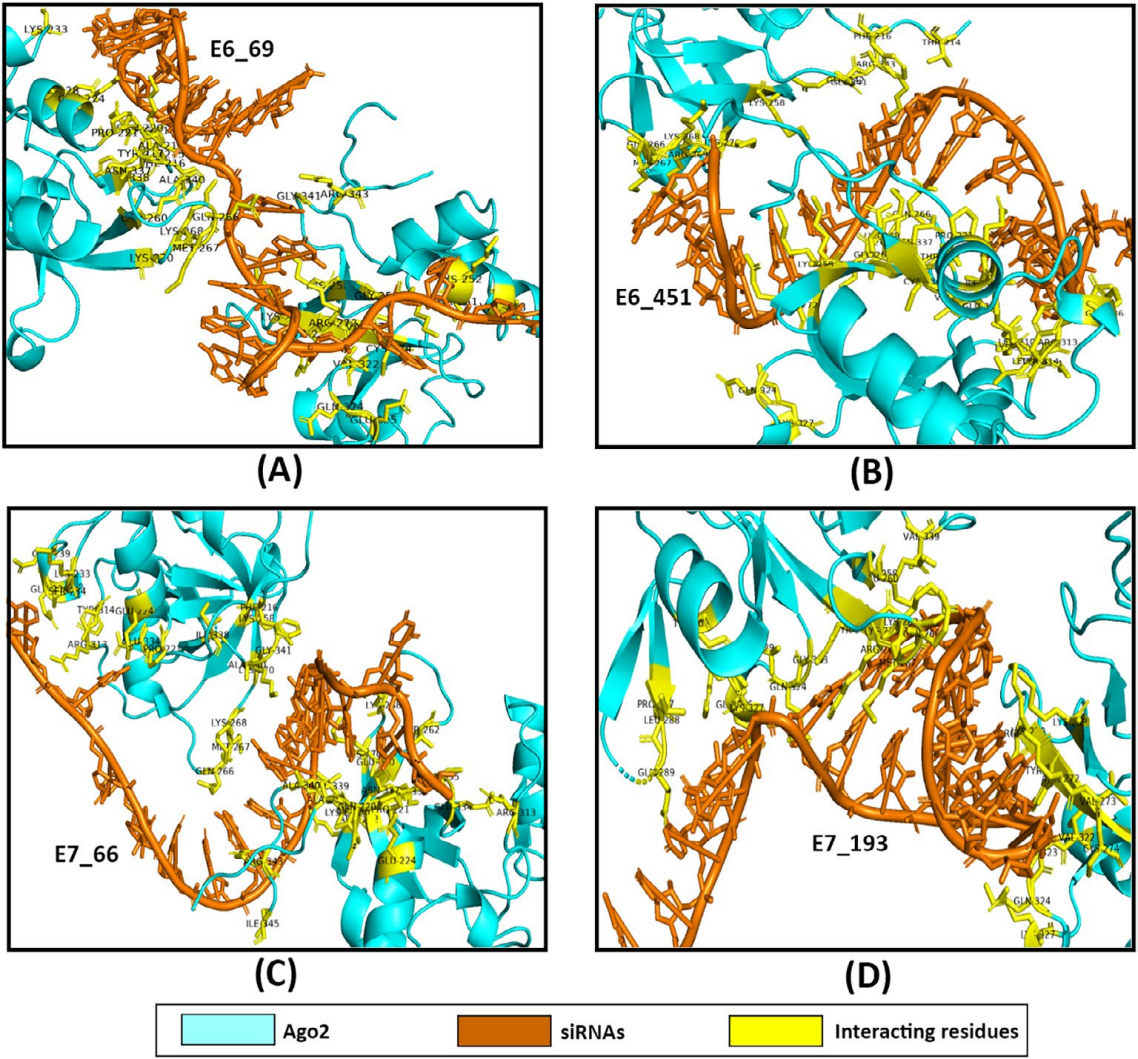
The protein‐siRNA interactions between (A) Ago2‐E6_69, (B) Ago2‐E6_451, (C) Ago2‐E7_66, and (D) Ago2‐E7_193.

**FIGURE 8:**
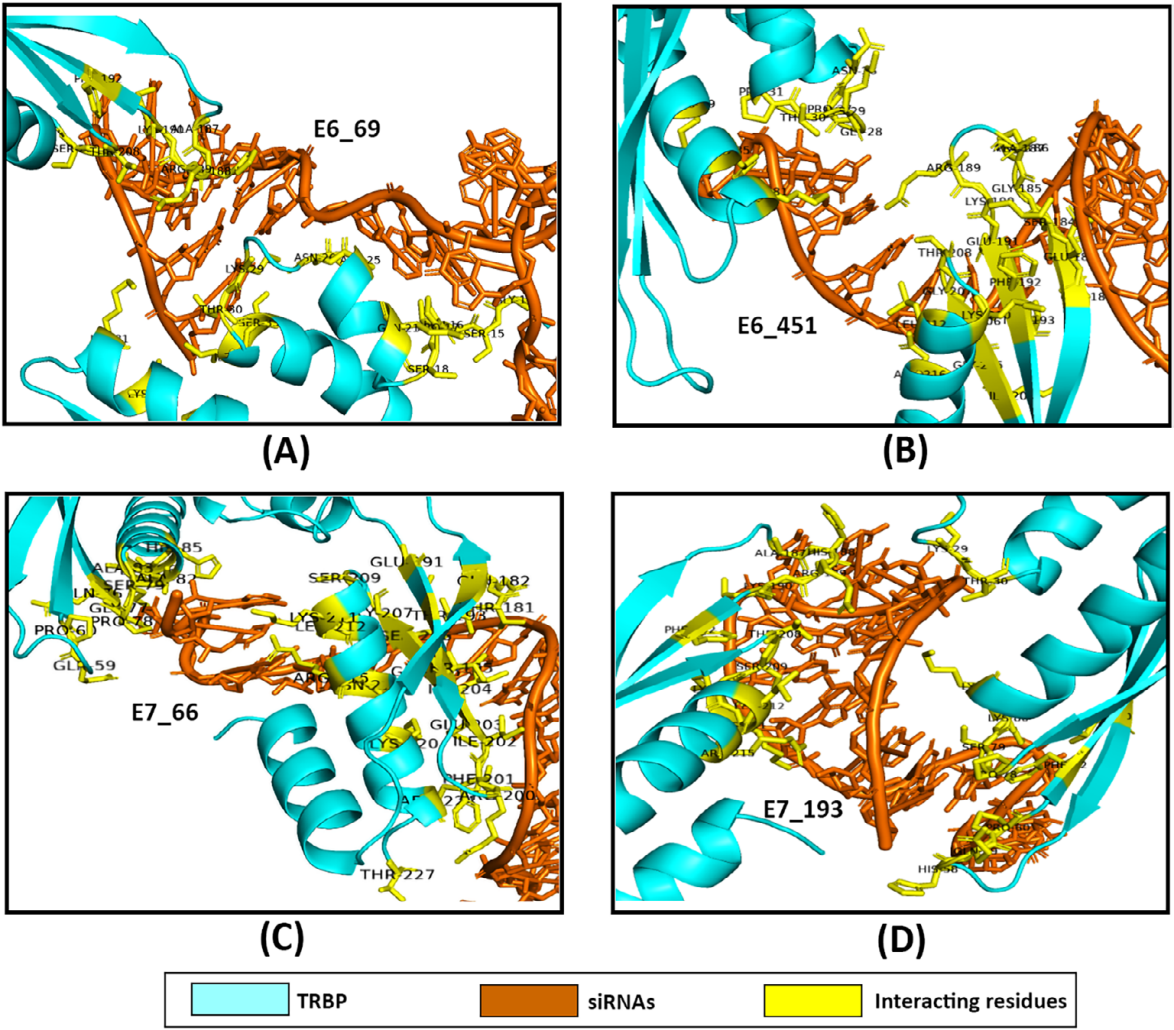
The protein‐siRNA interactions between (A) TRBP‐E6_69, (B) TRBP‐E6_451, (C) TRBP‐E7_66, and (D) TRBP‐E7_193.

The docking analysis revealed notable variations in the binding affinities and interaction profiles of the four siRNAs with the Dicer, Ago2, and TRBP proteins. Regarding the E6_69, it was found that the siRNA exhibited a significant binding affinity for Ago2, with a docking score of −282.64 kcal/mol. This was followed by the binding affinity towards TRBP (−270.53 kcal/mol) and Dicer (−258.04 kcal/mol) (Figure 5, Table 3). Further, the interacting residues between the siRNA and the proteins were found to be different. Therefore, E6_69 had different residual interactions with Dicer (9), Ago2 (6), and TRBP (5) (Figures 6A, 7A, and 8A). When compared to the control siRNA, E6_69 showed a strong binding affinity for Dicer, except for Ago2 and TRBP. Additionally, it exhibited a higher degree of common residual interactions with Ago2 (10) compared to Dicer (2) and TRBP (4). Among the residual interactions, E6_69 had the highest number of hydrogen bonds with Dicer (6), followed by Ago2 (4) and TRBP (2). Conversely, the siRNA had the highest number of van der Waals contacts with Dicer (10), followed by TRBP (7) and Ago2 (6) (Table 3).

**TABLE 3:** Docking results of four siRNAs interacting with Dicer, Ago2, and TRBP proteins.

siRNA	Docking score (Kcal/mol)	Interacting residues	Common interactions with the control	H‐bond	Van der Waals
Dicer‐E6_69	−258.04	ASN‐928, ASP‐930, HIS‐933, GLU‐954, LYS‐965, ARG‐1010, LYS‐1011, LYS‐1013, TRP‐1014	2	6	10
Ago2‐E6_69	−282.64	LYS‐252, LYS‐255, LYS‐258, ARG‐272, GLY‐323, ARG‐343	10	4	6
TRBP‐E6_69	−270.53	ASN‐26, SER‐33, HIS‐188, ARG‐189, LYS‐190	4	2	7
Dicer‐E6_451	−274.30	TYR‐842, GLU‐894, LYS‐906, GLU‐907, ARG‐925, ASP‐930, HIS‐933, TRP‐1014	0	5	7
Ago2‐ E6_451	−294.00	THR‐262, ARG‐272, LEU‐310, VAL‐311, ARG‐313, GLU‐334, ASN‐337	7	6	3
TRBP‐ E6_451	−251.67	GLY‐28, LYS‐29, PRO‐31, GLU‐183, SER‐184, ARG‐189, THR‐193, SER‐206, GLY‐207, ASN‐216	0	6	10
Dicer‐ E7_66	−309.20	LYS‐798, PRO‐801, ARG‐849, TYR‐866, VAL‐868, LYS‐885, PHE‐886, MET‐887, ARG‐927, ASN‐928, GLN‐931, HIS‐933, ARG‐934, PHE‐935, HIS‐982, SER‐985, LEU‐987, LYS‐1009, LYS‐1013, GLN‐1018, HIS‐1052	9	7	18
Ago2‐ E7_66	−306.00	ALA‐219, PRO‐221, GLU‐260, LYS‐270, ARG‐272, ASN‐337, GLU‐334, VAL‐339, ARG‐343	4	7	6
TRBP‐ E7_66	−249.21	PRO‐78, LYS‐86, ILE‐202, GLN‐182, SER‐206, LYS‐211, LEU‐212, ARG‐215, ASN‐216, ARG‐224	0	4	14
Dicer‐ E7_193	−304.19	PHE‐844, SER‐845, ARG‐849, TYR‐866, ARG‐927, ASN‐928, ASP‐930, GLN‐931, HIS‐933, PHE‐950, SER‐952, GLU‐954, TYR‐961, LYS‐965, TYR‐966, SER‐984, SER‐985, ARG‐986, TRP‐1014, GLN‐1021, ILE‐1022, LEU‐1023	8	11	23
Ago2‐ E7_193	−333.74	LYS‐258, GLN‐266, LYS‐268, ARG‐269, LYS‐270, TYR‐271, ARG‐272, GLN‐289, GLN‐324, LYS‐327, HIS‐328	14	15	14
TRBP‐ E7_193	−372.38	PHE‐62, PRO‐78, LYS‐80, LYS‐81, ALA‐187, HIS‐188, ARG‐189, LYS‐190, PHE‐192, SER‐ 209, LYS‐210, LYS‐211	10	6	16
Dicer_control	−251.81	PRO‐801, GLN‐802, GLU‐820, GLU‐821, TYR‐866, ARG‐849, TYR‐1049, HIS‐1052	—	2	10
Ago2_control	−330.00	LYS‐258, HIS‐263, ARG‐269, LYS‐270, ARG‐272, PHE‐286, TYR‐303, ARG‐307, HIS‐308, GLN‐324, LYS‐327, HIS‐328, TYR‐330	—	10	11
TRBP_control	−326.34	LYS‐44, VAL‐47, LEU‐51, LYS‐52, HIS‐58, ARG‐65, THR‐74, ALA‐82, LYS‐86, LYS‐211, ARG‐215, HIS‐226	—	4	26

With the lowest docking score of −294.00 kcal/mol, the E6_451 exhibited the highest affinity for Ago2, followed by Dicer and TRBP, with subsequent docking scores of −274.30 kcal/mol and −251.67 kcal/mol, respectively (Figure 5, Table 3). Moreover, the siRNA had 8, 7, and 10 residual interactions with Dicer, Ago2, and TRBP, respectively (Figures 6B, 7B, and 8B). However, the siRNA showed a greater affinity for Dicer than the control siRNA, except for Ago2 and TRBP. Subsequently, no shared residual interactions were found between Dicer and TRBP, although 7 shared residual interactions were found between siRNA and Ago2. Additionally, the siRNA had several intermolecular interactions with Dicer, Ago2, and TRBP proteins, including hydrogen and van der Waals bonds. Consequently, Dicer had 5 hydrogen bonds, while Ago2 and TRBP had 6 with siRNA. Besides, it had 7, 3, and 10 van der Waals interactions with Dicer, Ago2, and TRBP, respectively (Figure 5, Table 3).

The E7_66 exhibited the highest binding affinity for the Dicer, followed by the Ago2 and TRBP, with subsequent docking scores of −309.20, −306.00, and −249.21 kcal/mol, respectively (Figure 5, Table 3). The siRNA showed different residual interactions with the Dicer, Ago2, and TRBP as of 21, 9, and 10, respectively (Figures 6C, 7C, and 8C). Except for the Ago2 and TRBP, the E7_66 siRNA exhibited a higher binding affinity for the Dicer than the control siRNA. Importantly, no common residual interactions were found between the siRNA and TRBP, although 9 and 4 shared interactions with Dicer and Ago2, respectively. However, the siRNA forms hydrogen and van der Waals bonds with the Dicer, Ago2, and TRBP proteins. Thus, it had 7 hydrogen bonds with the Dicer and Ago2 each and 4 with the TRBP. It also had 18, 6, and 14 van der Waals interactions with the Dicer, Ago2, and TRBP, respectively (Figure 5, Table 3).

Additionally, the Dicer‐E7_193 had a docking score of −304.19 kcal/mol, indicating a strong binding affinity of the E7_193 to the Dicer. However, the siRNA showed the highest binding affinity to the Ago2 protein with a docking score of −333.74 kcal/mol, followed by the TRBP (−372.38 kcal/mol) (Figure 5, Table 3). There were 22, 11, and 12 residual interactions of the siRNA with the Dicer, Ago2, and TRBP, respectively (Figures 6D, 7D, and 8D). Importantly, the E7_193 showed a better binding affinity for the Dicer, Ago2, and TRBP proteins than the control siRNA. Also, the siRNA demonstrated the maximum number of common residual interactions compared to the control siRNA, including 8, 14, and 10 residual interactions within the E7_193‐Dicer, E7_193‐Ago2, and E7_193‐TRBP, respectively. In terms of residual interactions, the siRNA had the highest hydrogen bonds with the Ago2 (15), followed by Dicer (11) and TRBP (6). In contrast, the Dicer (23) had the maximum number of van der Waals interactions with the siRNA, followed by TRBP (16) and Ago2 (14) (Figure 5, Table 3).

## 4 Discussion

Through a multistep process, the normal cervical epithelium can lead to the formation of preneoplastic cervical intraepithelial neoplasia, which can eventually develop into invasive cervical cancer [63]. The functional inactivation of the TP53 and Rb tumor suppressor proteins by HPV‐derived E6 and E7 oncoproteins is considered a critical step in developing cervical cancer [28]. Considering the immense worldwide incidence of cervical cancer—especially in middle‐income and low‐income countries where death rates are disproportionately elevated—gene therapy represents a vital therapeutic strategy. Gene therapy offers a potential, precision‐based strategy by directly targeting the genetic drivers of the disease, such as HPV oncogenes, to enhance outcomes in cases where traditional therapies are limited or inaccessible [64]. Consequently, the blockade of the HPV oncogenes E6 and E7 is vital since they are recognized for their significant involvement in the progression of cervical cancer. RNAi has emerged as a new therapeutic approach and is now undergoing early‐stage clinical studies. RNAi is an innate mechanism by which specific mRNA molecules are broken down, suppressing genetic expression or gene silencing [65]. The first RNAi or miRNA gene, “lin‐4,” was identified in 1993 [65]. However, the mechanism of RNAi was elucidated in 2006, where it was found that gene silencing is mediated by dsRNA rather than single‐stranded antisense RNA [66]. On the other hand, siRNA is a double‐stranded RNA molecule, 21–23 nucleotides in length, that functions in a post‐transcriptional, sequence‐specific manner [65, 67, 68]. In 2018, the first siRNA‐based medication, “Patisiran” (Onpattro), was approved for treating transthyretin‐mediated amyloidosis, representing a major milestone in RNAi therapy [69].

Multiple studies have been conducted based on siRNA strategies targeting the HPV oncogenes, particularly concentrating on E6 and E7 transcripts, which are essential for viral oncogenesis. Most studies used chemically generated siRNAs for temporary knockdown, but a few utilized plasmid‐based or shRNA constructs for prolonged silencing [28]. Frequently used HPV‐positive cervical cancer cell lines include SiHa (HPV16) [2, 29, 70, 71, 72, 73, 74, 75, 76, 77, 78], CaSki (HPV16) [1, 2, 28, 29, 72, 74, 75, 76, 78, 79], and HeLa (HPV18) [1, 72, 74, 80, 81, 82, 83, 84], with a limited number of research exploring rarer lines such as SKG‐II/IIIa [81] and OM431 [84]. Several studies expanded these results to in vivo models, such as nude or nu/nu mice, in which siRNAs were delivered intratumorally or intravenously, often leading to substantial tumor growth suppression. Several studies have employed combinatorial approaches, such as co‐expression of siRNA with wild‐type TP53 or the simultaneous application of siRNA and cisplatin, thereby augmenting therapeutic effectiveness [28, 70, 71, 84]. However, initially, most studies confirmed transcript knockdown and antiproliferative effects in vitro; subsequent assessments progressed to in vivo testing and refined delivery methods. These data underscore the therapeutic potential of siRNA‐mediated silencing of E6/E7 in HPV‐associated malignancies, highlighting the importance of delivery mechanisms, target selectivity, and combination therapies in enhancing treatment efficacy [28]. Consequently, we designed four siRNAs using advanced bioinformatics techniques and algorithms, specifically targeting the high‐risk HPV strains, HPV 16 and HPV 18.

In the systematic design of efficient siRNAs, several sequence‐based factors and scoring algorithms are often used to improve silencing efficacy while minimizing off‐target effects. These parameters include GC content (> 40%) [39], Ui‐Tei rules (Ia or Ib) [40], Amarzguioui score > =3 [41], i‐Score > =66 [37], and Reynolds score > = 6 [39]. Together, these parameters provide a comprehensive framework for identifying effective siRNA candidates and enhancing the overall efficacy of RNAi research. A crucial parameter is the GC%, with an ideal range of 30%–52%, which maintains a balance between duplex stability and strand separation, promoting effective RISC loading [39]. The Ui‐Tei rules further guide siRNA functionality by recommending an A/U at the 5′ end of the antisense strand, G/C at the 5′ end of the sense strand, low internal stability at the 5′ antisense end, and the absence of internal repeats, all of which support accurate strand selection and potent gene silencing [40]. The Amarzguioui scoring system integrates positional nucleotide preferences and thermodynamic asymmetry, which is crucial for recognizing highly functional siRNAs [41]. Similarly, the i‐Score algorithm utilizes a regression model to assess nucleotide composition and duplex instability, providing a quantitative prediction of siRNA efficacy [37]. The Reynolds scoring system, often used in siRNA design, allocates points according to optimal sequence characteristics, including particular nucleotide positions, minimal internal repeats, and optimal GC content, with elevated scores associated with enhanced silencing efficacy [39].

In this study, the i‐Score Designer identified four potential siRNAs, and all of them met the suggested cutoff values of the aforementioned algorithms. Four siRNAs—E6_69, E6_451, E7_66, and E7_193—were selected based on their performance against different algorithm criteria: GC content > 40%, Ui‐Tei Ia or Ib, Amarzguioui > = 3, i‐score > =66, and Reynolds > = 6. Among the four selected siRNAs, the E6_69 exhibited a GC content of 52.6% and received high scores across all evaluated algorithms: a Reynolds score of 7, an i‐Score of 80.4, and an Amarzguioui score of 4 (Table 1). This siRNA demonstrated strong potential for gene silencing and fell under the Ui‐Tei Ia classification, indicating its potential in targeting the HPV's oncogenes [40]. Besides, the E6_451 had a GC content of 42.1% and also met the Ui‐Tei Ia classification. It showed a Reynolds score of 6, an i‐Score of 69.6, and an Amarzguioui score of 4, suggesting it might be a suitable candidate for effective HPV gene silencing [41]. Although the E7_66 had a GC content of 31.6%, below the preferred threshold of 40%, it still showed efficacy in gene silencing, with a Reynolds score of 9, an i‐Score of 73.2, and an Amarzguioui score of 3. Also, this siRNA was classified as Ia by the Ui‐Tei system, reinforcing its potential in gene silencing despite the lower GC content. Finally, the E7_193 exhibited a GC content of 47.4% and was categorized under the Ui‐Tei Ia classification. It provided a Reynolds score of 7, an i‐Score of 66.1, and an Amarzguioui score of 3, further establishing its candidacy for HPV's gene silencing [41].

The Tm for both sense and antisense strands of the siRNAs, determined by OligoEvaluator, was all below 65°C. Specifically, the E6_69 strands exhibited Tm values of 60.3°C and 62.9°C, while E6_451 had Tm values of 53.6°C and 59.3°C. For the E7_66, the Tm values were 48.0°C and 52.2°C, and for the E7_193, these were 56.1°C and 52.4°C (Table 2). These results suggest that the siRNAs possessed adequate thermal stability for effective silencing, as higher melting temperatures can enhance the binding affinity and specificity of siRNAs to their mRNA targets [39]. Furthermore, the RNAcofold analysis provided MFE scores and heterodimer ΔG values, indicating the stability of the siRNA‐mRNA complexes. The MFE scores ranged from −27.6 kcal/mol (E7_66) to −35.80 kcal/mol (E6_69), while the ΔG values ranged from −26.23 kcal/mol (E7_66) to −33.28 kcal/mol (E6_69) (Table 2). DINAMelt results further validated the stability of these siRNAs by providing detailed thermodynamic parameters [52]. The heat capacity values were 90.2°C, 81.9°C, 80.3°C, and 89.5°C for E6_69, E6_451, E7_66, and E7_193, respectively. The Tm values were similarly high, indicating strong duplex stability. Additionally, ΔG values of 50.2 kcal/mol (E6_69), 45.5 kcal/mol (E6_451), 40.3 kcal/mol (E7_66), and 46.5 kcal/mol (E7_193) suggested favorable free energy changes during duplex formation. The ΔH and ΔS values were also consistent with the formation of stable complexes, with ΔH ranging from 181.6 to 200 kcal/mol and ΔS ranging from 519.8 to 554.9 cal/mol/K (Table 2).

The stability and efficacy of siRNAs in gene silencing are influenced significantly by the folding free energies of both sense and antisense siRNA strands [40]. In this study, we utilized the MaxExpect tool to estimate these energies, revealing a range from 1.4 to 2.0 (Figure 2, Table 2). Notably, the antisense strands consistently demonstrated higher folding energies compared to their sense counterparts. This discrepancy suggested a greater structural complexity or a higher degree of secondary structure formation in antisense strands, which may affect their interaction with the RISC and, consequently, their gene‐silencing efficiency [40]. The antisense strand of E6_69 exhibited the highest folding energy (2.0), followed by E6_451 (1.9), E7_66 (1.9), and E7_193 (1.7) (Figure 2, Table 2). These values implied that the E6_69's antisense strand has the most stable secondary structure among the evaluated siRNAs, potentially contributing to its higher binding specificity and stability [40]. Complementary to the MaxExpect findings, the Duplexfold server provided detailed insights into the folding energies of both sense and antisense strands of the designed siRNAs [54]. The server's analysis indicated that the E6_69 had the highest free energy of folding and the highest folding accuracy with an energy of −36.3 kcal/mol. This substantial negative value suggested a highly stable folded structure, key for interacting with the RISC components and targeting mRNA recognition [54]. Alongside, the folding energies for the E6_451, E7_66, and E7_193 were predicted to be −32.5, −28.1, and −31.8 kcal/mol, respectively (Figure 3, Table 2). These differences in folding energies reflect variations in the stability and formation of secondary structures among the siRNAs. Higher negative values in folding energy indicate more stable duplex formations, which are essential for maintaining the integrity of the siRNA‐mRNA complex during gene silencing [54].

The RISC is integral to the process of siRNA‐mediated gene silencing and is crucial in siRNA‐based gene therapy. The essential elements of the RISC‐loading complex—Dicer, Ago2, and TRBP—facilitate the accurate processing, loading, and activation of siRNAs [85, 86]. Dicer, an RNase III endonuclease, processes long double‐stranded RNAs or precursor siRNAs into functional duplexes with approximately 21 long nucleotides featuring distinctive 2‐nucleotide 3′ overhangs [85, 87, 88]. TRBP serves as a cofactor that interacts with both Dicer and the siRNA duplex, facilitating the transfer of the duplex to Ago2 and enhancing the stability of the siRNA during the loading process [85, 89, 90]. Ago2, the essential component of the RISC complex, attaches to the guide strand of the siRNA and promotes the cleavage of matching target mRNA, thereby allowing for gene silencing [85, 91]. Effective interaction between siRNAs and these proteins is crucial for successful gene silencing and effective therapeutic outcomes. Molecular docking analysis between siRNAs and RISC components is now frequently used to assess binding affinities, identify interaction residues, and evaluate structural compatibility, thereby facilitating the identification of siRNAs that are more likely to be efficiently processed and loaded [92, 93].

In this study, the docking analysis revealed significant variations in the binding affinities and interaction profiles of the four siRNAs with the RISC proteins involved in the RNAi pathway: Dicer, Ago2, and TRBP. The E6_69 siRNA exhibited a notable binding affinity for Ago2 with a docking score of −282.64 kcal/mol, followed by the binding affinities for TRBP (−270.53 kcal/mol) and Dicer (−258.04 kcal/mol) (Figure 5, Table 3). When compared with the control siRNA, this E6_69 showed a stronger binding affinity for Dicer, and it showed a higher frequency of residual interactions with Ago2 (10) than with Dicer (2) and TRBP (4) (Figures 6A, 7A, and 8A). This siRNA also formed the highest number of hydrogen bonds with Dicer (6), suggesting a strong binding and specific interaction required for gene silencing [94]. Alongside, the E6_451 siRNA showed the highest binding affinity for Ago2 with a docking score of −294.00 kcal/mol, followed by Dicer (−274.30 kcal/mol) and TRBP (−251.67 kcal/mol) (Figure 5, Table 3). Compared to the control siRNA, the E6_451 exhibited a greater binding affinity for Dicer but lacked shared residual interactions with Dicer and TRBP, although it had 7 interactions with Ago2. However, it also exhibited numerous intermolecular interactions, including 5 hydrogen bonds with the Dicer and 6 with both Ago2 and TRBP, indicating a significant binding potential that could enhance its silencing efficacy (Figures 6B, 7B, and 8B) [94, 95, 96].

Besides, the E7_66 siRNA showed the highest binding affinity for the Dicer with a docking score of −309.20 kcal/mol, followed by the Ago2 (−306.00 kcal/mol) and TRBP (−249.21 kcal/mol) (Figure 5, Table 3). This siRNA also formed many intermolecular interactions, including 7 hydrogen bonds with Dicer and Ago2, and 4 with TRBP. Moreover, it exhibited a substantial number of van der Waals interactions: 18 with the Dicer, 6 with Ago2, and 14 with TRBP (Figures 6C, 7C and 8C). These extensive interactions suggested a strong binding capability, particularly with Dicer, a key component of RISC and its processing and gene‐silencing function [94, 96]. Furthermore, the E7_193 siRNA demonstrated strong binding affinity for the Ago2 with a docking score of −333.74 kcal/mol, followed by the Dicer (−304.19 kcal/mol) and TRBP (−372.38 kcal/mol) (Figure 5, Table 3). Notably, this siRNA showed the maximum number of shared residual interactions and hydrogen bonds with Ago2 (15), followed by Dicer (11) and TRBP (6). The van der Waals interactions were also significant, with 23 for the Dicer, 16 for the TRBP, and 14 for the Ago2 (Figures 6D, 7D and 8D). These extensive and varied interactions suggested that E7_193 had a strong binding affinity and stability, potentially making it the most effective siRNA for gene silencing among the four tested [94, 96]. The results of this study significantly enhance the credibility and potential utility of the siRNAs we have developed for targeted gene therapy. With a more sophisticated and comprehensive siRNA selection procedure, the current work distinguishes itself from other studies that often relied on standalone methodologies or focused on a specific HPV strain. This study lays the groundwork for additional promising treatment options by combining several computational approaches to develop siRNAs that target HPV‐16 and HPV‐18. This approach improves the accuracy and efficacy of siRNA design. It offers considerable potential for advancing future RNA‐based therapeutics, especially in addressing HPV‐related malignancies and enhancing the effectiveness of gene‐silencing treatments.

However, this study's findings require further assessment to determine the effectiveness of the selected siRNAs in other cell lines, either individually or in combination. This study also emphasizes the competency of using computer‐based siRNA design, selection, filtering, and evaluation to enhance the development of advanced therapeutic agents based on oligonucleotides. These agents are designed to combat HPV and provide prophylaxis for the global population.

## 5 Conclusion

The present study introduces an extensive computational framework to guide the design of siRNAs aimed at HPV oncogenes. The framework combines several established scoring algorithms—GC content, Ui‐Tei, Amarzguioui, i‐Score, and Reynolds—with sophisticated thermodynamic profiling (Tm, MFE, ΔG, ΔH, ΔS) and folding energy predictions through MaxExpect and DuplexFold. In this study, through rigorous computational approaches, we designed four siRNAs—E6_69, E6_451, E7_66, and E7_193—to ensure their potential efficacy in targeting HPV oncogenes (E6 and E7). Each siRNA was evaluated based on algorithmic criteria, including GC content, thermal stability, and binding affinity. The E6_69 siRNA was the most promising candidate, exhibiting immense potential for gene silencing, precise binding, and stable duplex formation. The thermodynamic and docking analyses further validated these siRNAs' stability and interaction profiles, respectively, particularly noting the high binding affinity and interaction strength of the E7_193 with RNAi RISC components. These results not only reinforce the potential of the selected siRNAs for HPV gene silencing but also underscore the effectiveness of computational tools in developing advanced oligonucleotide‐based therapeutic agents. However, this research does have certain limitations, such as the fact that we did not perform any in vivo or in vitro studies with the participants. Future research should focus on assessing these siRNAs in various cell lines, exploring alternative delivery methods, and optimizing transfection reagents. We hope this study can contribute to advancing targeted RNAi therapies and provide a promising avenue for combating cervical cancer globally.

## Author Contributions

**Md. Habib Ullah Masum:** conceptualization (lead), data curation (lead), formal analysis (lead), investigation (lead), methodology (lead), project administration (lead), resources (lead), software (lead), supervision (lead), validation (lead), visualization (lead), writing – original draft (equal), writing – review and editing (equal). **Rehana Parvin:** validation (equal), writing – original draft (equal), writing – review and editing (equal). **Homaira Pervin Heema:** writing – review and editing (supporting). **Aklima Akter:** writing – review and editing (supporting). **Jannatul Ferdous:** writing – review and editing (supporting).

## Funding

The authors have nothing to report.

## Ethics Statement

The authors have nothing to report.

## Conflicts of Interest

The authors declare no conflicts of interest.

## Data Availability

The authors have nothing to report.
